# Biomarkers in Systemic Sclerosis: An Overview

**DOI:** 10.3390/cimb45100490

**Published:** 2023-09-25

**Authors:** Giuseppe Di Maggio, Paola Confalonieri, Francesco Salton, Liliana Trotta, Luca Ruggero, Metka Kodric, Pietro Geri, Michael Hughes, Mattia Bellan, Michele Gilio, Selene Lerda, Elisa Baratella, Marco Confalonieri, Lucrezia Mondini, Barbara Ruaro

**Affiliations:** 1 Pulmonology Unit, Department of Medical Surgical and Healt Sciencies, Hospital of Cattinara, University of Trieste, 34149 Trieste, Italy; giuseppe.dimaggio@studenti.units.it (G.D.M.); metka.kodric@asugi.sanita.fvg.it (M.K.); pietro.geri@asugi.sanita.fvg.it (P.G.); lmondinifr@gmail.com (L.M.); 2 Division of Musculoskeletal and Dermatological Sciences, Faculty of Biology, Medicine and Health, The University of Manchester & Salford Royal NHS Foundation Trust, Manchester M6 8HD, UK; michael.hughes-6@manchester.ac.uk; 3 Department of Translational Medicine, Università del Piemonte Orientale (UPO), 28100 Novara, Italy; 4 Center for Autoimmune and Allergic Disease (CAAD), Università del Piemonte Orientale (UPO), 28100 Novara, Italy; 5 Department of Medicine, Azienda Ospedaliero–Universitaria, Maggiore della Carità, 28100 Novara, Italy; 6 Infectious Disease Unit, San Carlo Hospital, 85100 Potenza, Italy; 7 Graduate School, University of Milan, 20149 Milano, Italy; 8 Department of Radiology, Cattinara Hospital, University of Trieste, 34149 Trieste, Italy

**Keywords:** systemic sclerosis (SSc), autoimmune disease, interleukines, chemokines

## Abstract

Systemic sclerosis (SSc) is a complex autoimmune disease characterized by significant fibrosis of the skin and internal organs, with the main involvement of the lungs, kidneys, heart, esophagus, and intestines. SSc is also characterized by macro- and microvascular damage with reduced peripheral blood perfusion. Several studies have reported more than 240 pathways and numerous dysregulation proteins, giving insight into how the field of biomarkers in SSc is still extremely complex and evolving. Antinuclear antibodies (ANA) are present in more than 90% of SSc patients, and anti-centromere and anti-topoisomerase I antibodies are considered classic biomarkers with precise clinical features. Recent studies have reported that trans-forming growth factor β (TGF-β) plays a central role in the fibrotic process. In addition, interferon regulatory factor 5 (IRF5), interleukin receptor-associated kinase-1 (IRAK-1), connective tissue growth factor (CTGF), transducer and activator of transcription signal 4 (STAT4), pyrin-containing domain 1 (NLRP1), as well as genetic factors, including DRB1 alleles, are implicated in SSc damage. Several interleukins (e.g., IL-1, IL-6, IL-10, IL-17, IL-22, and IL-35) and chemokines (e.g., CCL 2, 5, 23, and CXC 9, 10, 16) are elevated in SSc. While adiponectin and maresin 1 are reduced in patients with SSc, biomarkers are important in research but will be increasingly so in the diagnosis and therapeutic approach to SSc. This review aims to present and highlight the various biomarker molecules, pathways, and receptors involved in the pathology of SSc.

## 1. Introduction

Systemic sclerosis (SSc) is a rare connective tissue disease, characterized by complex and different pathogenetic pathways, including vasculopathy, abnormal immune activation, with the production of autoantibodies and fibrosis [1,2,3,4]. The pathogenesis of SSc is complex and not yet completely understood, however the events involved in SSc pathogenesis can be schematically summarized in three main phases: (a) vascular damage, mainly of microcirculation; (b) immune system activation/autoimmunity/inflammation and (c) fibrosis [4,5,6,7,8,9,10]. Fibrosis of the skin and internal organs may be considered the main clinical hallmark of the disease and it is due to fibroblasts activation and dysfunction with the acquisition of a “myofibroblast phenotype” [1,2,11,12,13,14,15,16,17,18]. Skin manifestations can be recognized and studied by using the modified Rodnan skin score (mRss), the validated method for assessing the severity of skin involvement in SSc and distinguishing the different subsets of skin involvement in SSc [6,7,8,9,10]. The use of the mRss provides an assessment of skin thickness and has been used as a primary outcome measure in most clinical trials. The original score was developed in 1979 by Rodnan et al. [8]. In summary, these are the two main forms into which SSc can be formally classified at present: 1—limited cutaneous form (lcSSc), characterized by predominantly distal skin thickening and the presence of anticentromere antibodies, and 2—diffuse cutaneous form (dcSSc), in which there are diffuse distal and proximal skin changes associated with the presence of anti-topoisomerase antibodies, anti-RNA-III polymerase antibodies, or other antibodies with antinuclear pattern [9,10,11,12,13,14,19,20,21,22,23,24,25]. The most used biomarkers in SSc are autoantibodies, and in this case, they have utility for diagnosis, classification, and prognosis of the disease; it should also be mentioned that biomarkers can be potential therapeutic targets [4,23,24,25,26,27,28,29,30]. Antinuclear antibodies (ANA) are present in more than 90% of SSc patients, along with anticentromere, anti-Th/To, and anti-topoisomerase I antibodies, which are considered the classic biomarkers present in 60% of SSc patients and help to define precise clinical classifications [4,7,29,30,31,32,33,34,35,36,37]. Other autoantibodies, such as those directed against the endothelium or fibroblasts, against angiotensin II type 1 receptor, endothelin-1 type A receptor, platelet-derived growth factor receptor (anti-PDGFR), and extracellular matrix (ECM) proteins [16,17,18,19,20,38,39,40,41,42,43,44,45] are also found in SSc patients. More complex autoantibody systems against G-protein-coupled receptors, growth factors, and respective receptors have also been described in SSc [21,45,46,47,48,49]. ANA might not only represent biomarkers of disease but also play a pathogenic role through immune-mediated mechanisms and molecular mimicry. ANA (particularly anti-topoisomerase-I and anti-RNA polymerase III antibodies) appear to be transported within the cell by direct interaction with intercellular components and receptors, targeting intracellular topoisomerase and RNA polymerase by the corresponding antibodies [22,23,49,50,51,52,53,54,55].

Supporting this evidence is the response of some SSc patients to B-cell-targeted therapies and the role of activated B cells in the success of allogeneic bone marrow transplantation for the treatment of SSc [24,25,55,56,57,58,59,60]. In addition, SSc patients have macro- and microvascular damage and impaired peripheral blood perfusion [6,48,49,50,51,52,53]. Nail fold video capillaroscopy (NVC) plays an important role because it is a noninvasive and reliable method to detect microvascular involvement. NVC is now formally used to classify SSc because it plays a diagnostic role in recognizing microvascular involvement [3,4,26,60,61,62,63,64,65,66,67,68]. Indeed, the presence of capillaroscopic abnormalities of the nail fold, together with the presence of ANA and recurrent Raynaud’s phenomenon, are predictive factors for progression to definitive SSc. Anti-topoisomerase I antibodies are also predictive, in the first 3 years of disease of the development of diffused skin involvement and digital ulcers (DU), as well as severe interstitial lung disease (ILD) [66,67,68,69,70,71,72,73,74,75,76,77].

Anti-centromere autoantibodies (ACA) are associated with pulmonary arterial hypertension (PAH), anti-topoisomerase I autoantibodies (anti-topo I) with ILD, and anti-ti-RNA polymerase III autoantibodies (anti-RNA Pol III) with scleroderma renal crisis (SRC). Anti-RNA polymerase III autoantibodies may be a biomarker that predicts the rapid progression of skin thickening, gastric antral vascular ectasia, SSc-associated tumors, scleroderma renal crisis, and possibly autoimmune syndromes associated with silicone breast implants [7,27,28,48,49,50,51,52,53,77,78,79,80,81,82,83,84] (Table 1). 

However, SSc patients may also present with other autoantibodies, such as anti-PMScl (often associated with inflammatory muscle involvement), anti-Th/To, anti-RNP, and anti-fibrillarin autoantibodies [26,27,28,29,30]. Approximately 10% of SSc patients are ANA-negative, but both ANA-negative and ANA-positive patients may present with novel antibodies, including anti-elF2B, an-ti-RuvBL1/2 complex, anti-U11/12 RNP, anti-U3RNP, anti-BICD2, anti-Ku and an-ti-PM/Scl [7,8,9,10,84,85,86,87,88,89,90,91]. It is unusual to find two different SSc-specific autoantibodies simultaneously in the same individual [29,30,31,32,33,48,49,50,51,52,53,54,55,92,93,94,95,96,97,98,99]. Mortality in SSc patients is significantly increased and usually related to the life-threatening manifestations of SSc, including PAH, ILD, SRC, cardiac involvement, tumors, and infections, which are also related to immunosuppressive drugs [8,9,34,48,56,57,58,100,101,102,103,104,105,106,107,108,109].

The purpose of this review is to present an overview the various molecules, pathways, and biomarker receptors involved in SSc pathology.

## 2. Systemic Sclerosis Pathogenesis

The exact processes of SSc pathogenesis are not clear. However, the pathogenic processes can be summarized in three main events: the endothelial injury with a consequent microvasculopathy, followed by the autoimmune response and inflammation and finally a diffuse fibrosis of skin and internal organs [1,2,3,4,5,6,7,8,9,10,109,110,111,112,113,114,115,116,117,118]. Several studies confirmed the role of each of these three events and their interaction in SSc pathogenesis. However, the initial trigger event has not yet been identified. Recent studies supported the role of a genetic predisposition and both endogenous and/or exogenous environmental triggers may be promoter for epigenetic mechanisms in genetically predisposed population [1,2,3,4,5,6,7,8,9,10,68,69,70,71,72,73,74,75]. Interestingly, a recent study supported the role of infectious triggers in SSc pathogenesis; in particular, the authors reported a possible role of Parvovirus B19 in this disease [15]. 

### 2.1. The Role of Endothelial Injury in Systemic Sclerosis Pathogenesis

Regarding the endothelial injury, several studies reported the presence of an endothelial involvement before the development of fibrosis and this situation support the vascular origin of SSc [6,7,8,9,10,11,12,13]. Raynaud’s phenomenon (RP) represents the clinical expression of vasomotor instability and tendency to vasospasm [6,7,8,9,10,11,12,13,106,107,108,109]. The alteration of microvascular tone may represent a trigger leading to the opening of endothelial junctions increasing the vessel permeability and recalling inflammatory cells [6,7,8,9,10,11,12,13,22,23,24,25,106,107,108,109]. The increase of vessel permeability could be considered responsible for oedema that is present in the early phase of the disease and is correlated with the presence of puffy fingers [6,7,8,9,10,11,12,13,106,107,108,109]. The endothelium is an active tissue with important biologic functions, as the production of vasodilators (i.e., nitric oxide (NO) and prostacyclin) and vasoconstrictors (i.e., endothelin-1 (ET-1) and plated activating factor) and cell adhesion molecules [10,13]. In SSc vasculopathy may assume both destructive (loss of capillaries) and proliferative (hypertrophy of the vessel tunic) characteristics with a consequent decrease in vascular bed and ischemic suffering of skin and internal organs. The SSc vascular damage is characterized by the presence of a characteristic processes with a vasomotor instability with an imbalance of vasoactive factors: overproduction of vasoconstrictors (ET-1) and underproduction of vasodilators (NO and prostacyclin) [6,7,8,9,10,11,12,13,22,23,24,25,106,107,108,109]. The free radical nitric oxide (NO) is a potent vasodilator and is synthesized from L-arginine by NO synthase (NOS) [10,11,12,13,14,15,16,17,18,19,22,23,24,25,40,41,42,43,44,45,46,47,48,106,107,108,109,110,111,112,113]. Endothelial isoforms of NOS (eNOS or NOS 3) has been identified with a constitutive expression, other isoforms are shown in other cell types. Furthermore, an inducible expression (iNOS or NOS 2) in response to a variety of stimuli is possible, with NO-mediated signalling apparent in the skin [10,11,12,13,14,15,16,17,18,19,40,41,42,43,44,45,46,47,48,106,107,108,109,110,111,112,113]. Several studies reported that NO have a biphasic effect in physiological and pathological conditions, being both beneficial and detrimental depending on the concentration and local environment [10,11,12,13,14,15,16,17,18,19,40,41,42,43,44,45,46,47,48]. Recently observation proposed the regulation of NO by endogenous levels of the NOS inhibitor asymmetric dimethylarginine (ADMA) [10,11,12,13,14,15,16,17,18,19,22,23,24,25,40,41,42,43,44,45,46,47,48,106,107,108,109,110,111,112,113]. Regarding the endothelial dysfunction in SSc, there is evidence of reduced intracellular eNOS production in the SSc endothelium and increased endothelial activation [10,11,12,13,14,15,16,17,18,19,22,23,24,25,40,41,42,43,44,45,46,47,48]. Increased endothelial apoptosis mediated by anti-endothelial cell antibodies and antibody-dependent cell cytotoxicity has also been shown to precede inflammatory events and fibrosis [10,11,12,13,14,15,16,17,18,19,22,23,24,25,40,41,42,43,44,45,46,47,48]. While the aetiology of endothelial dysfunction is still unclear, free-radical-mediated damage and immunological insults remain attractive proposals to mediate effects. An interesting study suggested that SSc serum significantly reduces NO synthase activity, paralleled by decreases in intracellular cGMP and NO production in the cell medium, and supported the presence of a factor that inhibits NOS [10,11,12,13,14,15,16,17,18,19,22,23,24,25,40,41,42,43,44,45,46,47,48,106,107,108,109,110,111,112,113]. These results support the observations from earlier studies that serum factors influence endothelial dysfunction and apoptosis; however, it has been reported that SSc serum alone was ineffective and the additional presence of natural killer cells was required to mediate cytotoxicity [10,11,12,13,14,15,16,17,18,19,40,41,42,43,44,45,46,47,48,106,107,108,109,110,111,112,113]. In the same work it is also demonstrated that SSc serum alone had no significant effect on endothelial cytotoxicity and the observations of reduced NO activity and production were unlikely to be due to decreased cell survival. While free-radical-related mechanisms and immunological insults evidently mediate endothelial dysfunction, the observations of some papers also suggest the role of circulating inhibitory factors such as ADMA that may regulate NOS activity in vivo, and account for the observed serum induced decrease in cGMP and NO production in vitro [10,11,12,13,14,15,16,17,18,19,22,23,24,25,40,41,42,43,44,45,46,47,48]. In summary, formation of NO is increased in patients with primary RP or lcSSc, but nitration of proteins and ADMA is a particular feature of dcSSc and may reflect abnormal NO regulation and/or contribute to endothelial dysfunction in SSc [10,11,12,13,14,15,16,17,18,19,22,23,24,25,40,41,42,43,44,45,46,47,48].

Furthermore, endothelial cells presented an increased expression of adhesion molecules (VCAM1, ICAM and E-selectin) contributing to the recall of inflammatory cells from the circulation [6,7,8,9,10,11,12,13,22,23,24,25,106,107,108,109]. ET-1 also presents proliferative and fibrogenic effects and may contribute not only to the early response with a rapid and transient cell contraction but also to a slower process culminating in the remodeling phenotype of blood vessels [6,7,8,9,10,11,12,13,106,107,108,109]. This later response also involves perivascular fibroblasts and smooth muscle cells proliferation leading to the hypertrophy of intimal and medial layers and adventitial fibrosis of vessel. These processes cause a progressive obliteration of blood vessels contributing to a chronic ischemia, that together with endothelial cells apoptosis lead to a progressive loss of capillaries in the late phase of SSc [6,7,8,9,10,11,12,13,106,107,108,109]. In SSc patients, vascular endothelial growth factor (VEGF) is overexpressed leading to vascular malformations including giant and bushy capillaries, observed at NVC evaluation [6,7,8,9,10,11,12,13,106,107,108,109]. In SSc, the lack of new functional blood vessels is also due to an impaired vasculogenesis [6,7,8,9,10,11,12,13,106,107,108,109]. In fact, endothelial progenitors seem decreased in SSc patients leading to an incompetent vasculogenesis. In addition, pericytes, the vascular mural cells, contribute to the regulation of vascular development and remodeling, and they seem to be activated from the early phase of the disease inhibiting angiogenetic processes and being responsible to deposition of extracellular matrix (ECM) [6,7,8,9,10,11,12,13,106,107,108,109]. It is important to underline that the stimulation of ECM production and the accumulation of ECM proteins worsen tissue hypoxia resulting in a vicious circle of hypoxia and fibrosis [6,7,8,9,10,11,12,13,22,23,24,25,106,107,108,109].

### 2.2. The Role of Immune System in Systemic Sclerosis Pathogenesis 

The activation of immune system appears to be an early event, in SSc pathogenesis [6,7,8,9,10,11,12,13,106,107,108,109]. Perivascular infiltrates are mainly composed of T cells, but also macrophages, mast cells and B lymphocytes can be observed [6,7,8,9,10,11,12,13,40,41,42,43,44,45,46,47,106,107,108,109]. An abnormal immune response is characterized by the activation of T lymphocytes from the earlier phases of SSc. Although their exact role remains to be clarified, T cells seem to be involved both in the early inflammatory response and in the late fibrotic process with the production of cytokines leading to the recall of other cells as macrophages and mast cells. Recently, a new study confirmed an alteration of T-lymphocyte subset and of their serum cytokines in SSc patients confirming the role of T cells in the pathogenesis of the disease [6,7,8,9,10,11,12,13,44,45,46,106,107,108,109]. In SSc, among T cells, type 2 T helper (Th2) cells are more expressed than Th1 cells [6,7,8,9,10,11,12,13,40,41,42,43,44,45,106,107,108,109]. In the last decade, many studies focused on the role of T reg cells that in SSc have been described reduced in levels and with a decreased functional capacity [6,7,8,9,10,11,12,13,106,107,108,109]. T cells seem to contribute to the SSc progression through the secretion of several fibrogenic cytokines and chemokines [6,7,8,9,10,11,12,13,44,45,46,106,107,108,109]. IL-4 and IL-13 are released by Th2 cells and promote fibrosis, in fact IL-4 seems able to stimulate fibroblasts proliferation, migration and collagen production [6,7,8,9,10,11,12,13,106,107,108,109]. Contrarily, antifibrotic cytokines (IFN-γ, characteristic of Th1, and IL-10, characteristic of Treg) are decreased, indirectly contributing to fibrogenesis process in SSc. B cells represent a relatively small subpopulation of perivascular lymphocytes, however the presence of specific antibodies in almost all SSc patients, suggests a certain role of B cells in SSc pathogenesis. The exact mechanisms that stimulate the activation of B cells and the induction of a humoral immune response remain to be understood. There are several hypotheses, including molecular mimicry, the potential expression of autoantigen peptides and a B cells hyperactivity due to intrinsic B cells abnormalities [6,7,8,9,10,11,12,13,106,107,108,109]. The contribution of the SSc specific antibodies to the disease development remains to be clarified; however, recent data suggest a possible role of antibodies on endothelial damage with the interaction with growth factor (PDGF) receptors and ET-1 receptor [6,7,8,9,10,11,12,13,106,107,108,109]. In SSc pathogenesis, the role of B cells is not confined to the production of antibodies, in fact activated B lymphocytes may also contribute to fibrosis process stimulating fibroblast through the IL-6 pathway [6,7,8,9,10,11,12,13,106,107,108,109]. 

### 2.3. The Role of Fibrotic Process in Systemic Sclerosis Pathogenesis 

The increased production and deposition of ECM is the most prominent hallmark of SSc affecting both skin and internal organs. ECM consists of different proteins, as collagens, proteoglycans, fibronectin and adhesion molecules [6,7,8,9,10,11,12,13,42,43,44,45,46,47,48,106,107,108,109]. Collagen is the most important component of ECM. Collagen is produced by fibroblasts that can be considered the key effectors of fibrosis in SSc, in fact, they presented an activated phenotype in myofibroblasts and both the early endothelial dysfunction and the innate immune system activation are crucial in the fibrotic process [6,7,8,9,10,11,12,13,106,107,108,109]. In addition, recent studies described a relative resistance to apoptosis of fibroblasts in SSc [6,7,8,9,10,11,12,13,106,107,108,109]. The TGFβ stimulation is considered responsible for the activation of fibroblasts. However, it is not able to explain all the phenotypic characteristics of these cells in SSc. TGFβ is secreted by different cells (platelets, monocytes/macrophages, T cells, and fibroblast) and it is not the only fibrogenic mediators in SSc, also PDGF and CTGF (connective tissue growth factor) are considered leading actors in the pro-fibrotic process. PDGF is a mitogen and chemoattractant for fibroblasts, released by endothelial cells, platelets and macrophages. PDGF seems able to increase the secretion of TGFβ and IL-6 and to stimulate collagen, fibronectin and proteoglycan production. CTGF may stimulate fibroblasts growth and promote the synthesis of part of ECM [6,7,8,9,10,11,12,13,40,41,42,43,44,45,106,107,108,109].

Furthermore, an abnormal pro-fibrotic Th2- polarized T cell response has been proposed to mediate tissue damage and fibrosis in SSc-ILD as Th2 cytokines lead to the activation of alternative inflammatory pathways and to the transcription of transforming growth factor (TGF)-β, involved in induction and progression of fibrosis [8,9]. Moreover, it has been demonstrated that IL-4/IL-13 axis upregulate genes known to be involved in the mechanisms of wound healing and fibrosis in murine models and in vitro, infuencing the activation of myofbroblasts [2,3,4,5,6,7,8,40,41,42,43,44,45,46]. Several studies reported that Th2 cytokines contribute to the differentiation and migration of eosinophils, which are involved in systemic inflammatory process in SSc patients [2,3,4,5,6,7,8,40,41,42,43,44,45,46]. At a later stage, fibroblasts proliferation and activation lead to extracellular matrix deposition and fibrosis of the lung parenchyma [2,3,4,5,6,7,8,40,41,42,43,44,45,46].

Recent studies shown that alterations in macrophage polarisation are involved among the possible immune system abnormalities contributing to SSc pathogenesis. Macrophages have been classified as classically (M1) or alternatively (M2) activated, although growing evidence indicates that they may exhibit characteristics shared by more than one of the described phenotypes, in particular an M2 pre-eminent phenotype has been postulated for SSc monocytes/macrophages [2,3,4,5,6,7,8,40,41,42,43,44,45,46]. Several recent studies demonstrated that M2 and more significantly cells expressing both M1 and M2 surface markers identified patients with SSc in comparison of the healthy population; on the contrary, they observed no differences when only M1 markers were used. The authors of numerous paper demonstrated that the initial gating strategy based on the CD204+cells resulted the most efficient to describe monocyte/macrophage phenotype differences between SSc patients and healthy subjects [2,3,4,5,6,7,8,40,41,42,43,44,45,46]. 

Furthermore, the result of other works supported the above observations demonstrating a remarkable plasticity of circulating monocytes/macrophages, resulting in a ‘spectrum’ of activation states and other papers showed a downregulation of interferon-γ response and IL6/JAK/STAT3 pathway in SSc monocyte-derived macrophages, possibly describing a ‘SSc specific macrophage’ [2,3,4,5,6,7,8,40,41,42,43,44,45,46].

## 3. Biomarkers in Systemic Sclerosis 

Recent studies have shown how vast and ever-expanding the field of biomarkers is in SSc. Biomarkers will become increasingly important in research and, consequently, in the diagnosis and therapeutic approach to SSc [30,31,32,33,34,35,36,59,60,61,62,63,64,65,66,67]. Several studies have demonstrated the involvement of at least 240 pathways and numerous dysregulated proteins in the pathogenesis of SSc, so the field of biomarkers in this disease is complex and evolving [30,31,32,33,34,35,36,59,60,61,62,63,64,65,66,67]. Cytokeratin 17 (CK17), marginal zone protein B1 (MZB1), and leucine-rich α2-glycoprotein-1 (LRG1) appear to be potential biomarkers for SSc, with CK17 negatively associated with disease severity and higher values of CK17 protective [37,59,60,61,62,63,64,65,66,67]. A potential therapeutic target could be endostatin, which is associated with vascular manifestations in SSc and is specifically elevated in progressive SSc; it has been considered a marker of disease severity [38,62,63,64,65,66,67].

Periostin is secreted by fibroblasts and epithelial cells and is associated with cell adhesion, fibrosis, angiogenesis, survival and matrix remodeling [34,39,40,41]. In SSc, circulating periostin levels are elevated and associated with disease duration, skin fibrosis, and cardiomyopathy [39,40,41]. The chemokine CC2 (CCL2) also appears to be involved in the development of fibrosis in SSc [39,40,41,42,62]. MicroRNAs (miRNAs) are short nucleotide sequences involved in cellular regulation [37,38]. The miR-138 and miR-27a microRNAs suppress major pathways involved in epithelial-to-mesenchymal cell transition and subsequent fibrosis [37,38,45,60,61,62,63,64,65,66,67]. The relative expression of miR-138 and miR-27a is significantly lower in SSc patients than in controls, while only miR-138 is further depressed in diffuse cutaneous SSc, thus potentially both could be used as diagnostic biomarkers with miR-138 specific to predict severity [45,60,61,62,63,64,65,66,67]. Suppression of carcinogenicity receptor 2 (ST2) binds IL-33 and serum soluble ST2 (sST2) suppresses IL-33 signaling [45,60,61,62,63,64,65,66,67]. Elevated serum sST2 levels are associated with increased joint disease activity and increased hand dysfunction in SSc, indicating that sST2 could be a biomarker to predict SSc joint involvement [45,60,61,62,63,64,65,66,67]. Angiopoietins (Ang-1 and Ang-2) interact with the specific receptor tyrosine kinase Tie2 to modulate endothelial cell activation, vascular remodeling, and angiogenesis [45,60,61,62,63,64,65,66,67]. In SSc patients, Ang-1 decreased while Ang-2 increased, thus this may contribute to both vascular ablation and new abnormal blood vessel formation [41,42,43,44,45,60,61,62,63,64,65,66,67]. In their interesting study Michalska-Jakubus et al. suggested for the first time that dysregulation of angiopoietins/VEGF system with shift towards Ang2 and VEGF and decrease in Ang1 levels may play a role in progression of SSc specific microangiopathy from capillary enlargement and collapse to aberrant vessel repair and final loss of angiogenesis [12]. The authors reported that VEGF levels seem to be increased early in SSc but in face of attenuated Ang1 it might lead to ectatic microvessels of increased permeability as reflected by giants and microhaemorrhages typical for an “Active” NVC pattern. The researcher supported that elevated levels of VEGF appeared to be sustained also in advanced stages of microangiopathy and correspond with capillary loss (“Late” NVC pattern) [12]. In conclusions, the results of this study might be partially explained by the relative advantage of Ang2 that increase later in disease progress and result in advanced vascular damage. These eminent researchers identified that altered Ang1/Ang2 profile might underlay loss of angiogenesis in SSc despite increase in VEGF and may be one of the essential factors promoting SSc specific microangiopathy. Moreover, since Ang1 is an “endothelial survival factor”, it is possible that its relative insufficiency in SSc patients, as revealed in this article, might be the mechanism underlying dysfunction and damage of endothelial cells thought to be a primary event in disease pathogenesis [12].

The IFN-regulated protein sialic acid-binding Ig-like lectin 1 (SIGLEC-1) is upregulated in SSc compared with controls but is not associated with specific complications [45,60,61,62,63,64,65,66,67]. Increased activation and expression of type 1 interferons are typical of SSc and may provide a potential therapeutic target [45,60,61,62,63,64,65,66,67]. In SSc, the lack of new functional blood vessels is also due to impaired vasculogenesis [2,7,29]. Indeed, endothelial progenitors appear to be reduced in SSc patients, leading to incompetent vasculogenesis. Furthermore, pericytes, which are mural cells, contribute to the regulation of vascular development and remodeling and appear to be activated from the early stage of the disease by inhibiting angiogenic processes and being responsible for the deposition of extracellular matrix (ECM) [2,7,29]. Abnormal accumulation of ECM usually occurs in SSc [2,29]. ECM catabolism is regulated by matrix metalloproteinases (MMP-1 to MMP-28) whose activity is in turn inhibited by tissue MMP inhibitors (TIMP-1 to TIMP-4) [2,29]. TIMP-4 is increased in SSc patients compared to healthy subjects [2,29]. Similarly, semaphorins (Sema3A-F) have anti-angiogenic effects and are increased in SSc patients [2,29]. Cytokines and interleukins (e.g., IL-1, IL-4, IL-6, IL-13, IL-17B, IL-17E, IL-17F, IL-22, IL-35) and chemokines (e.g., CCL2,3,5,20,21) are often elevated in SSc [30,31,32,33,34,35,36,37,38,59,60,61,62,63,64,65,66,67] (Table 2).

In the following sections, the role of disease-relevant biomarkers of specific organ systems in SSc will be discussed.

## 4. Biomarkers in Systemic Sclerosis Interstitial Lung Disease 

Interstitial lung disease (ILD) is one of the most common manifestations of SSc (SSc-ILD), affecting approximately 40–60% of patients, and pulmonary hypertension (PH) is the leading cause of both mortality and disability in SSc [69,70,71,72,73,74,75]. Pulmonary involvement is responsible for approximately 35% of all SSc-related deaths [70,71,72,73,74,75,76,77].

Risk factors associated with the development of SSc-ILD include male gender, diffuse cutaneous SSc, African American heredity, and the presence of anti-Scl-70 (anti-topoisomerase I) antibodies [77,78,79,80,81,82,83,84]. The clinical course, timing of onset, and spectrum of severity of ILD vary among patients with SSc, making the diagnosis and management of ILD very difficult. Indeed, in many subjects with SSc pulmonary involvement remains limited and stable even without treatment [79,80,81,82,83,84,85,86,87]. Conversely, some patients may present with severe and/or rapidly progressive ILD and for this reason, many efforts have been made to identify patients at risk for ILD and its greater severity. Among these were the dcSSc subset, African-American race, older age and disease onset, shorter disease duration, positive abs anti-Mouse I, and absence of ACA [86,87,88,89,90,91,92,93,94].

Lung endothelial damage is central to the pathogenesis of ILD. Histologically, SSc-ILD presents as a picture of nonspecific interstitial pneumonia, unlike idiopathic pulmonary fibrosis, which is usually characterized by the usual picture of interstitial pneumonia [93,94,95,96,97,98]. SSc-ILD is characterized by inflammation early in the disease, extensive endothelial dysfunction, and increased deposition of ECM, particularly collagen produced by activated myofibroblasts in resident tissues [79,80,81,82,83,84,85,86,87]. As ECM increases, lung tissue stiffness increases, leading to restrictive lung disease with reduced lung compliance, decreased lung volumes, and diffusing capacity, resulting in decreased exercise tolerance, dyspnea, fatigue, hypoxia, hypertension lung disease, work disability, and shortened life. expectation. The disease process is thought to be initiated by repetitive epithelial and endothelial cell injury with activation of the immune system, recruitment of fibroblasts, and phenotypic transformation of the fibroblast into a myofibroblast which then secretes excessive ECM resulting in fibrosis [84,85,86,87,88,89,90]. The initial endothelial and epithelial lesions are probably autoimmune and inflammatory in nature but could also be induced by pathogens and environmental factors [88,89,90,91,92,93,94]. In some epithelial cells, the apoptotic process takes place, stripping the alveoli, while other epithelial cells mutate into myofibroblasts with reduced apoptosis, loss of polarity, increased migration, and increased production of ECM, including collagen [88,89,90,91,92,93,94]. The reduced apoptotic capacity of myofibroblasts can cause an abnormal persistence of these active cells, contributing to progressive fibrosis [81,82,83,84,85,86,87]. Transforming growth factor b (TGF-β) is fundamental in the process of accumulation of ECM and therefore fibrosis, as well as dysregulation of the immune system towards inflammation [81,82,83,84,85,86,87]. Stimulation of TGFβ is thought to be responsible for the activation of fibroblasts, however, it is not able to explain all the phenotypic characteristics of these cells in SSc. For example, when stimulated by TGFβ, fibroblasts acquire a myofibroblast phenotype, however, this process requires other events such as co-expression of the variant form of fibronectin extra domain (ED-A) [81,82,83,84,85,86,87]. In addition, epithelial cells, pericytes, and bone marrow-derived cells can differentiate into myofibroblast-like cells. TGFβ is secreted by various cells (platelets, monocytes/macrophages, T cells, fibroblasts) and is not the only fibrogenic mediator in SSc. PDGF and CTGF (connective tissue growth) are also considered to be major players in the fibrotic process. CTGF can stimulate the growth of fibroblasts and promote the synthesis of part of the ECM. PDGF is a mitogen and chemoattractant for fibroblasts, released by endothelial cells, platelets, macrophages and fibroblasts. It seems able to increase the secretion of TGFβ and IL-6 and stimulate the production of collagen, fibronectin and proteoglycans [81,82,83,84,85,86,87]. Other biomarkers typically implicated in SSc-ILD include specific autoantibodies, signal transducer and activator of transcription 4 (STAT4), CD226 (DNAX accessory molecule 1), interferon regulatory factor 5 (IRF5), associated interleukin-1 kinase-1 to the cell receptor (IRAK1), connective tissue growth factor (CTGF), pyrin-containing domain 1 (NLRP1), T-cell surface zeta-chain glycoprotein (CD3ζ) or CD247, the NLR family, SP-D (surfactant protein), KL-6 (Krebs von den Lungen-6), IL-8, leucine-rich α2-glycoprotein-1 (LRG1) and CCL19, as well as genetic factors including the -DRB1 allele [81,82,83,84,85,86,87]. SSc-ILD was particularly associated with anti-topoisomerase I antibodies (anti-Scl-70 antibody), antinuclear antibodies with nucleolar pattern (including anti-RNA-polymerase III, anti-NOR-90 anti-Th/To, anti- PM/Scl-75, anti-U3-RNP/fibrillarin or anti-PM/Scl-100 antibodies) [81,82,83,84,85,86,87]. Anti-PM/Scl defines SSc patients with a high frequency of ILD, calcinosis, dermatomyositis skin changes, and severe myositis [81,82,83,84,85,86,87]. The biomarkers most associated with active lung disease and progression specifically in SSc-ILD are KL-6 (Krebs von den Lungen-6), SP-D (surfactant protein), C-reactive protein, and CCL19 although other biomarkers are also present non-specific can be elevated [81,82,83,84,85,86,87]. Th2 lymphocytes produce IL-13 and IL-4 which stimulate fibroblasts and activate pro-fibrotic macrophages M2 which induce TGF-β, plaque-derived growth factor (PDGF), and fibroblast growth factors (FGF) which induce the activation of myo-fibroblasts [81,82,83,84,85,86,87]. Biomarker chemokines including CCL18, CX3CL1 and CXCL with and without RNA complexes have recently been associated with SSc-ILD [81,82,83,84,85,86,87].

In the progression of SSc-ILD, an important role is played by IL-6, secreted by myofibroblasts, M1 macrophages, and B lymphocytes, which increases the expression of IL-4 and IL-13 receptors, enhancing macrophage polarization M2 and increasing fibrosis [81,82,83,84,85,86,87]. Tocilizumab acts by inhibiting the IL-6 receptor, consequently decreasing the activation of myofibroblasts and reducing the polarization of M2 macrophages; this underlines the antifibrotic effect of this molecule and supports the central role of IL-6 [81,82,83,84,85,86,87]. B cell activation is also common in SSc, and B cells increase a number of angiogenic factors [81,82,83,84,85,86,87]. B-cell depletion suppresses pro-fibrotic macrophage differentiation and thereby inhibits fibrosis, providing the rationale for anti-B-cell agents such as rituximab in SSC-ILD [81,82,83,84,85,86,87]. Among the biomarkers mentioned above, autoantibodies and C-reactive proteins are the only biomarkers typically used in routine clinical practice. Anti-Scl-70 antibodies are associated with an increased incidence of progressive SSc ILD [81,82,83,84,85,86,87]. Additional autoantibodies against anti-phosphatidylinositol-5-phosphate 4-kinase type 2 beta (PIP4K2B) and AKT serine/threonine kinase 3 (AKT3) have been linked to increased pulmonary fibrosis in SSc (114). However, various other biomarkers are under investigation for clinical use, including KL-6 (Krebs von den Lungen-6), CCL18 (chemokine [C-C motif] ligand 18), MMP7 (matrix metalloproteinase-7), MMP12 (matrix metalloproteinase-12), IL-6, CXCL4 (chemokine ligand 4 [C-X-C motif]), CXCL3 (chemokine ligand 4 [C-X-C motif]), and chitinase-3-like protein 1 (YKL-40) as recently reviewed [81,82,83,84,85,86,87]. The presence of MMP-12 has been shown to be increased in SSc-ILD compared to SSc forms without ILD and correlates with the degree of pulmonary fibrosis [81,82,83,84,85,86,87]. Sirtuins are NAD-dependent protein deacetylases that regulate angiogenesis; SIRT1 and SIRT3 correlate with the degree of pulmonary fibrosis in SSc [81,82,83,84,85,86,87]. The chemokine CCL2 is increased in SSc and predicts the long-term progression of SSc-ILD [81,82,83,84,85,86,87]. Jee et al. recently described a composite biomarker index consisting of SP-D, Ca15-3 and ICAM-1 that identifies SSc-ILD [84,85,86,87,88,89,90]. Cold inducible RNA binding protein (CIRP) has also been associated with SSc-ILD and may be responsible for disease activity and response to therapy [81,82,83,84,85,86,87].

**Figure 1:**
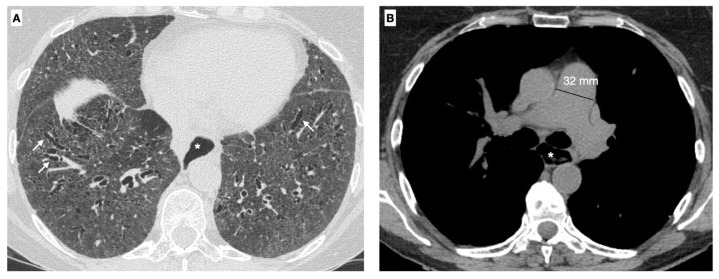
A 63-year-old female with the diagnosis of SSc. (**A**) Axial High-resolution CT scan shows traction bronchiectasis (white arrows) in a back-ground of diffuse ground-glass opacities, related to a fibrotic nonspecific pneumonia (NSIP) patter. (**B**) Using the mediastinal window setting the dilatation of the main pulmonary artery (32 mm) can also be recognized as sign of pulmonary hypertension, a common complication of the disease. Moreover a dilatation of the esophagus (*) can be seen (operator E.B., Radiology Unit, University of Trieste, University Hospital of Cattinara).

Histopathologically, SSc-ILD is characterized by an early pulmonary infiltration of inflammatory cells into the lung parenchyma and can be classified into specific disease patterns, including non-specific interstitial pneumonia (NSIP), habitual interstitial pneumonia (UIP), organized pneumonia and lymphoid pneumonia [83,84,85,86,87,88,89,90]. Among the different patterns of ILD, the most common pattern in SSc is NSIP (50-77% of SSc patients with ILD) with bilateral involvement typically starting at the bases. The UIP pattern occurs more rarely during SSc and is characterized by shorter survival than NSIP. SSc-ILD is detected after diagnosis by high resolution CT (HR-CT) and progression by both HR-CT and pulmonary function tests (PFT) (Figure 1) [95,96,97,98]. Studies have shown how quantitative CT scanning can be used to detect early SSc-ILD and differentiate it from interstitial pneumonia and to follow the progression of SSc-ILD more precisely [95,96,97,98,99].

**Figure 2:**
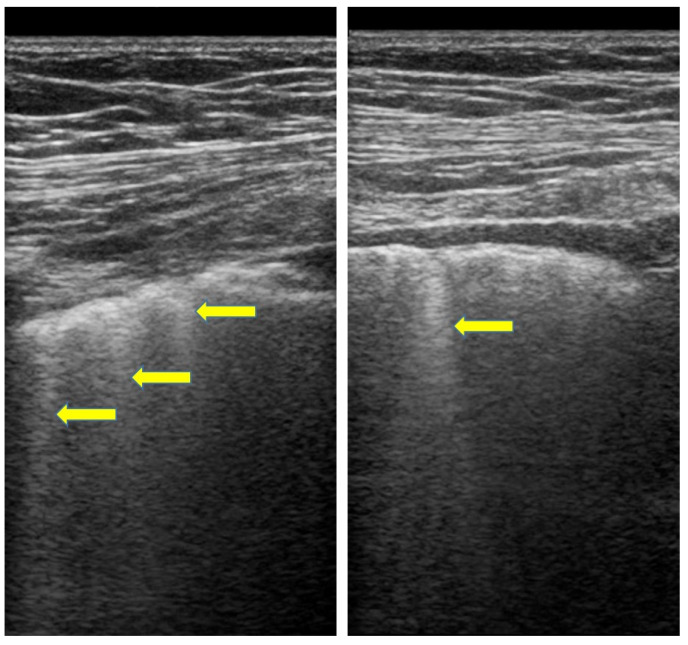
LUS in SSc. Advanced ILD (B-lines, yellow arrows) using a linear probes (7.5 MHz, operator F.S., Pulmonology Unit, University of Trieste, University Hospital of Cattinara).

Recently lung ultrasound (LUS) in SSc patients highlighted the role of this technique in the assessment of ILD. Given ultrasound radiation-free nature and the possibility to perform this examination at bedside the use of this technique in ILD evaluation has become increasingly popular in the last decades [95,96,97,98,99]. Through the identification of B-lines, vertical hyperechogenic lines arising from pleural line, LUS may help to discover signs of lung interstitial syndrome [97,98,99] (Figure 2). 

Significant or progressive SSc-ILD usually requires therapy [6,88,89,95,96,97,98,100,101,102,103,104,105,106,107,108]. The PFT-based OMERACT (outcome measures in rheumatic diseases) detects progression of SSc-ILD defined as a ≥10% decline in forced vital functions (FVC) or ≥5% to <10% decline in FVC with a decline relative ≥15% DLCO [84,87,99,100,101,102,103,104,105]. Importantly, with effective therapy for SSc-ILD, pulmonary fibrosis is stabilized or the rate of decline in lung function is reduced [84,87,101,102,103,104,105,106]. Thus, emphasis has accumulated on early diagnosis and timely therapy of SSc-ILD before significant irreversible lung damage occurs. The drugs typically used to treat SSc-ILD are non-specific immunosuppressants (cyclophosphamide, mycophenolate), more specific immunosuppressive drugs including anti-IL-6 agents (tocilizumab) and anti-B-cell drugs (rituximab), and antifibrotics agents (nintedanib—a tyrosine kinase inhibitor) [6,88,89,95,96,97,98,101,102,103,104,105,106,107,108]. The non-specific immunosuppressants, mycophenolate, an inhibitor of guanosine nucleotide synthesis, and cyclophosphamide, an alkylating agent, decrease the proliferation of fibroblasts, T helper cells and B cells and thus have significant anti-fibrotic effects [6,88,89,95,96,97,98,100,101,102,103,104,105,106,107,108]. Indeed, mycophenolate is currently considered the standard and baseline therapy for SSc-ILD. Tocilizumab is increasingly used for SSc-ILD. In particular, the antifibrotic effect associated with the action of Tocilizumab is linked to the inhibition of the IL-6 receptor, which determines the activation of myofibroblasts and the reduction of the M2 macrophage polarization [6,88,89,95,96,97,98,101,102,103,104,105,106,107,108]. In SSc-ILD, anti-B cell agents, such as Rituximab, are used with the rationale that B cell depletion suppresses pro-fibrotic macrophage differentiation and consequently inhibits fibrosis [6,88,89,95,96,97,98,100,101,102,103,104,105,106,107,108]. Nintedanib, a tyrosine kinase inhibitor, is an antifibrotic drug that inhibits PDGF, FGF and vascular endothelial growth factor (VEGF) receptors, reducing fibrosis [6,88,89,95,96,97,98,100,101,102,103,104,105,106,107,108]. However, among antifibrotics, pirfenidone appears to have less beneficial effects in SSc, so it has no indication in the treatment of this condition [6,88,89,95,96,97,98,100,101,102,103,104,105,106,107,108]. Among treatments, autologous hematopoietic stem cell transplantation (AHSCT) is an alternative for rapidly progressive forms of SSc unresponsive to these agents or for early SSc-ILD; on the other hand, lung transplantation is the alternative for end-stage lung disease [6,88,89,95,96,97,98,100,101,102,103,104,105,106,107,108]. In conclusion, multiple arms of the immune system are activated in SSc-ILD providing many candidate biomarkers and potential therapeutic targets.

## 5. Biomarkers in Systemic Sclerosis Vascular Injury, Focus on Pulmonary Arterial Hypertension

The most important clinical manifestation of vascular disease in SSc is Raynaud’s phenomenon (RP) and digital ischemia [6,88,89,95,96,97,98,109,110,111,112,113,114,115]. RP is a variable and complex symptom; it represents a vasospastic condition and is characterized by an initial vasoconstriction/occlusion of pre-capillary arterioles (pallor/white phase), followed by a cyanotic phase (purple phase), and finally by post-ischemic hyperaemia (red phase). Cold exposure and emotional stress are the main triggers of RP. It is often the presenting symptom of the disease and consists of reversible and transient tissue ischemia, but in SSc it can be persistent resulting in the formation of digital ulcers (DU) and/or gangrene [6,88,89,95,96,97,98,109,110,111,112,113,114,115]. Digital ulcers are characterized by significant pain and reduced quality of life with hand-related disability [6,88,89,95,96,97,98,110,111,112,113,114,115,116]. In addition, DUs are burdened with fearsome complications, such as infection and osteomyelitis.

**Figure 3:**
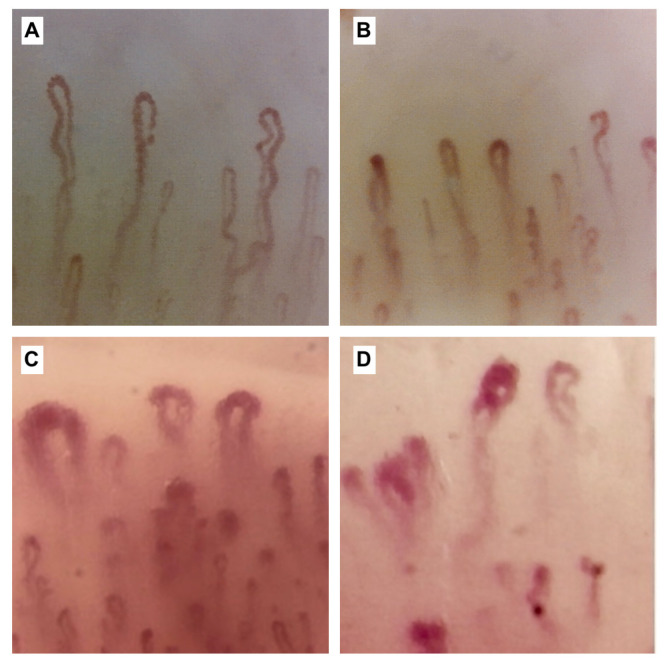
Nailfold capillaroscopy images (×200). Figure (**A**) shows the capillaroscopic findings in a healthy subject with capillaries of normal number and shape, i.e., the so-called U-shaped and hairpin-like morphology; (**B**–**D**) show the capillaroscopic changes in the scleroderma pattern in the early (**B**), active (**C**) and late (**D**) phases, respectively and associated with a progressive distortion of the architecture of normal capillaries, which gradually deviate from the A pattern. In particular in figures (**B**,**D**), megacapillaries (defined as capillaries >50 μm) and an increase (**B**) and a subsequent decrease (**C**) in capillary density, respectively, are observed. The later picture (**D**) is characterised by a further accentuation of these anomalies, with a greater decrease in capillary density and an additional number of giant capillaries. In (**B**), there are also areas of microhaemorrhages, observable in the early phase but also present in the active phase (operators L.M. and B.R, Pulmonology Unit, University of Trieste, University Hospital of Cattinara).

Among instrumental examinations, NVC is a noninvasive technique to assess capillary morphology and architecture (Figure 3). As described above, NVC is also included in the 2013 ACR/EULAR classification criteria. Capillaroscopic abnormalities of SSc include enlarged (giant) capillaries, microhemorrhages, and capillary leakage [10]. Note that these changes can also be detected in other connective tissue diseases, although they are more frequent in SSc. RP is followed by telangiectasias, ischemic DU, pitting scars, periungual microvascular abnormalities, pulmonary arterial hypertension (PAH), and cardiac disease affecting function and exercise tolerance [6,88,89,95,96,97,98,110,111,112,113,114,115,116]. All are considered outcomes of vascular damage in SSc.

**Figure 4:**
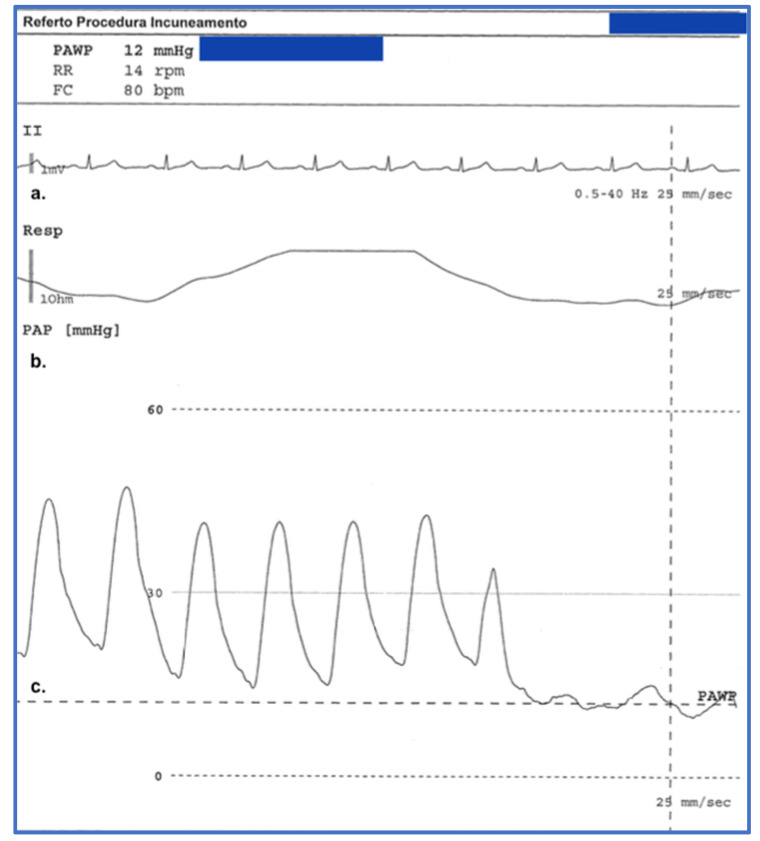
Graph obtained from a right heart catheterisation procedure performed at our centre in a 68-year-old patient with Systemic Sclerosis with pulmonary involvement and interstitial disease. Pulmonary hypertension is in fact one of the complications of pulmonary involvement by systemic sclerosis and affects about 12% of patients with this rheumatological disease. This graph shows on the y-axis respectively the ECG monitoring (derivation VII) which is performed throughout the procedure (a), in the picture (b) it is also possible to observe the measurement of the pulmonary arterial pressure (PAP) and the end-expiratory pulmonary artery wedge pressure (PAWP) in the picture (c), (operator P.G., Pulmonology Unit, University of Trieste, University Hospital of Cattinara).

Pulmonary hypertension (PH) is hemodynamic condition, defined by a mean pulmonary arterial pressure (mPAP) > 20 mmHg at rest. PAH (pulmonary arterial hypertension) is characterized by a mean pulmonary arterial pressure (mPAP) greater than 20 mmHg and pulmonary arterial wedge pressure (PAWP) less than 15 mmHg on right heart catheterization (RHC) (Figure 4) with a PVR > 3 WU in the absence of significant interstitial lung disease (ILD) [116,117,118,119]. PAH is due to vasculopathy of the small and medium calibre pulmonary arteries (pulmonary arterial hypertension) which leads to vascular remodelling and to increase in pulmonary vascular resistance (PVR). PAH occurs in 7% to 19% of SSc patients depending on the population and duration of the disease [116,117,118,119,120,121]. 

The diagnosis of PAH is often made at a late stage, as in the early stages’ patients may be asymptomatic or complain of nonspecific symptoms. Therefore, patients with SSc should be screened for PAH, even if asymptomatic, by cardiac echocolordoppler, respiratory testing, and NT-ProBNP assay. Diagnostic algorithms have been proposed to identify patients for RHC, which is still the gold standard tool for diagnosing PAH and PH [116,117,118,119,120,121,122,123,124,125,126]. Risk factors for PAH include severe Raynaud’s phenomenon, severe digital ischemia, cutaneous telangiectasias, chronic disease, late onset of the disease, advanced age, postmenopausal status, reduced diffusing capacity (DLCO < 50%), DLCO/alveolar volume less than 70%, forced vital capacity/DLCO less than 1.6, and increased right ventricular systolic pressure greater than 2 mmHg/year [110,111,112,113,114,115,116,117,118,119].

Screening should include specific autoantibodies (anti-topoisomerase I (SCL-70), an-ti-centromere and anti-RNA polymerase III and antiphospholipid antibodies), pulmonary function tests, echocardiography, pro-terminal brain natriuretic peptide (NT-proBNP), capillaroscopy of nail folds and initial high-resolution CT scan to rule out ILD, and, in case of PAH, right heart catheterization to determine PA pressure [118,119,120,121,122,123,124,125,126,151]. Many molecules have been associated with vascular complications of SSc, so there are many potential biomarkers for vascular disease and PAH in SSc. Activation, endothelial cell apoptosis, specific autoantibodies, infectious agents, reactive oxygen species, and other causes that provide many potential biomarkers may contribute to vascular lesion formation [121,122,123,124,125,126,151]. Once activated, endothelial cells secrete endothelin-1 (ET-1), von Willebrand factor (vWF), nitric oxide, and endothelial nitric oxide synthase, resulting in unstable vascular tone, with decreased vasodilation and increased vasoconstriction causing ischemia and tissue hypoxia [119,120,121,122,123,124]. Endothelin-1 stimulates fibroblasts to convert to activated myofibroblasts with increased ECM secretion, intimal hyperplasia, luminal narrowing, reduced capillary blood flow vessel obliteration and ischemia [120,121,122,123,124,125,126,151].

Local secretion of the von Willebrand factor causes platelet aggregation, hypercoagulability, and fibrin deposition leading to terminal vascular injury [126,127,128,129,130,131,132,133,151]. The endothelial-mesenchymal transition also favors the formation of myofibroblasts [128,129,130,131,132,133,152,153]. The activated endothelium also expresses an increase in adhesion molecules and specific chemokines, recruiting immune cells and perivascular infiltrates leading to further inflammation and fibrosis [128,129,130,131,132,133,152,153]. Specifically, the imbalance of cytokines, including endothelial growth factor (VEGF), matrix metalloproteinase (MMP)-9, endoglin, endothelin-1 (ET-1), and the angiostatic pentraxin 3 (PTX3), MMP-12, endostatin, angiostatin and semaphorin3E (Sema3E) cause dysfunction in angiogenesis and an alteration in endothelial progenitor cell (EPC) recruitment [116,117,118,119]. Endothelin-1 is secreted by endothelial cells and activated smooth muscle cells, fibroblasts, epithelial cells, and inflammatory cells [128,129,130,131,132,133,152,153]. An increase in ET-1 is present in SSc-PAH compared to SSc patients without PAH and healthy controls [116,117,118,119]. It was demonstrated that ET-1 levels were reduced by bosentan treatment in SSc patients with PAH to the levels present in SSc patients without PAH, indicating that this biomarker could be an indicator of the severity of vascular damage and response to treatment. bosentan therapy [154,155,156]. Pentraxin 3 is a receptor produced by activated endothelial cells, macrophages, smooth muscle cells, dendritic cells, and fibroblasts [116,117,118,119,138,154,157,158,159,160,161,162,163,164,165,166,167,168,169,170,171,172,173,174,175,176,177,178,179,180,181,182,183]. However, pentraxin 3 levels have only variable associations with vasculopathy in SSc [116,117,118,119]. Endostatin is an angiostatic peptide that blocks VEGF activity and has been associated with PAH, scleroderma renal crisis, and cardiac involvement [116,117,118,119]. Angiostatin antagonizes several growth factors, including VEGF, and is elevated in patients with more advanced vascular disease in SSc [116,117,118,119]. Matrix metalloproteinases destroy the ECM and MMP-9 levels were decreased in SSc-PAH and increased with bosentin therapy, while MMP-12 was increased in patients with DU and NVC changes [116,117,118,119]. TGF-β remains important for all manifestations of SSc including PAH [116,117,118,119]. Endoglin (CD105) is an accessory receptor for TGF-β and higher circulating endoglin is related suggesting that this was a biomarker for vascular damage in SSc [116,117,118,119]. Thrombomodulin (TM), CD163, and NT-proBNP are elevated in SSc-PAH [116,117,118,119]. High levels of maresin 1 are associated with the development of DU in SSc [116,117,118,119]. Elevated asymmetric dimethylarginine (ADMA) is an endogenous inhibitor of nitric oxide (NO) that affects endothelial function and is elevated in microvascular disease in SSc [116,117,118,119]. Hypochromic erythrocytes have been closely associated with the prognosis of SSC-PAH [116,117,118,119]. Hemoglobin and ferritin are significantly lower in patients with pulmonary hypertension (PH) in SSc than in those with pulmonary hypertension, while uric acid and NT-proBNP are significantly higher [116,117,118,119]. CCL21 circulating in SSc is a biomarker associated with PAH and the development of PAH [128,129,130,131,132,133,152,153]. SSc-PAH is associated with elevated metalloproteinase inhibitors, including TIMP-4 levels, indicating a cardiopulmonary vasculature-specific role of TIMP-4 activation in SSc [116,117,118,119]. Neuropilins (NRP1-2) are non-tyrosine kinase glycoprotein receptors expressed on endothelial cells and are potential predictive biomarkers of PAH, nail fold capillary abnormalities, and DU [116,117,118,119]. Sirtuins (SIRT1-7) NAD-dependent protein deacetylases that regulate angiogenesis; SIRT1 and SIRT3 are decreased in SSc and microvascular disease, and SIRT3 is specifically related to the presence of DU [116,117,118,119]. Slit glycoproteins (Slit1-3) are implicated in angiogenesis and are increased in SSc and patients with microvascular disease (150). Carcinoembryonic antigen-related cell adhesion molecule (CEA-CAM)-positive monocytes are associated with inflammation and ILD in SSc patients [116,117,118,119]. Circulating levels of IL-18 are higher and SSc correlates positively with PAH [116,117,118,119]. Similarly, IL33 and soluble carcinogenicity suppression 2 (ST2), are increased in SSc, especially with DU and PAH [128,129,130,131,132,133,152,153]. IL-32 and macrophage migration inhibitory factor (MIF) are elevated in SSc patients with PAH [116,117,118,119]. The chemokines CCL20, CCL21, and CCL23 are also elevated in SSc-PAH [116,117,118,119]. Recently elevated CXCL4 levels and decreased CXCL5 levels have also been associated with SSc-DU [116,117,118,119]. Similarly, the chemokines CXCL16 and GDF15 are elevated in SSc-PAH [116,117,118,119]. CX3CL1 (fractalkin) is elevated in SSc with DU (68). Resistin is increased in SSc with DU and in SSc-PAH [116,117,118,119]. Galectin-3 was also higher in SSc patients with DU [116,117,118,119]. Adipsin, visfatin, interferon-gamma and type 1 interferons are increased in SSc-PAH [116,117,118,119]. Aptamic proteomics of serum exosomes define patterns that could distinguish primary Raynaud’s disease from early SSc and, with RNA networks, are potential biomarkers for vascular disease in SSc [116,117,118,119].

Treatment of PAH involves a stepwise approach using single agents or combination therapy with phosphodiesterase type 5 (PDE-5) inhibitors (including sildenafil and tadalafil), soluble guanylate cyclase (sGC) stimulators (including riociguat), endothelial receptor antagonists (including bosentan ambrisentan, and macitentan), prostacyclin analogs (epoprostenol, treprostinil, and iloprost), or selective prostacyclin IP receptor agonists (selexipag), supported by anticoagulants, diuretics, digoxin, and calcium channel blockers where appropriate [116,117,118,154,155,156].

## 6. Biomarkers in Systemic Sclerosis Skin Involvement

As previously reported, among the hallmarks of SSc, skin involvement is one of the pivotal symptoms and is used to classify SSc patients in routine clinical practice into three different subgroups, limited skin involvement (lcSSc), diffuse skin involvement (dcSSc), and limited SSc (lSSc) [1,2,3,4,5]. Although in different sizes and except for patients with SSc sine Scleroderma (ssSSc), the skin is always involved in SSc. SsSSc is a very rare subgroup characterized by the complete or partial absence of skin involvement but with the presence of internal organ involvement and typical serologic abnormalities.

The modified Rodnan skin score (mRss) is the validated method to assess the severity of skin involvement in SSc and to distinguish, as mentioned above, patients with lcSSc from those with dcSSc or with lSSc [1,2,3,4,5,6,7,8,9].

The mRss is a sum of ratings obtained from clinical palpation of 17 skin areas (cheekbone, fingers, back of hands, forearms, arms, chest, abdomen, thighs, legs, and feet) [11,12,13]. Skin thickness is assessed by palpation and scored on a scale ranging from 0 (normal), 1 (weak), 2 (intermediate) to 3 (severe skin thickening).

**Figure 5:**
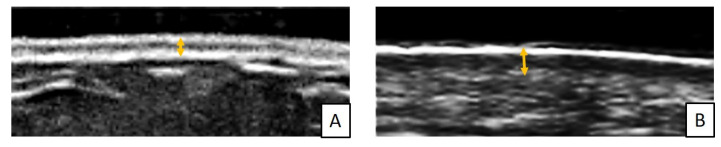
Example of measurement of dermal thickness (yellow arrows) by skin high-frequency US (18 MHz probe) in a healthy subject (**A**) and in an SSc patient (**B**) at the level of arm (operator L.R, Pulmonology Unit, University of Trieste, University Hospital of Cattinara).

The mRss has some disadvantages, for example, it cannot detect small but clinically relevant changes in skin thickness over time. Recently, several studies have reported the usefulness of high-frequency skin ultrasound (US) for early identification of skin involvement in patients with SSc [6,7,8,9]. Most of the authors used a US equipped with a probe at a frequency of 10–30 MHz, as high frequencies are needed to study skin thickness to achieve good resolution, even if penetration is poor. This allows good visualization that can distinguish the epidermis, dermis, and subcutaneous fat, providing a thickness determination and qualitative assessment of the skin; the authors made a comparison with mRss, the current gold standard for the study of skin compromise in patients with SSc [17] (Figure 5).

Skin involvement in SSc patients has a great impact on patients’ quality of life as it can cause pruritus, depigmentation, edema, traction ulcers, and movement difficulties; however, it is not associated with increased mortality. Skin involvement is initially due to edema caused by microvascular lesions and inflammation, subsequently to increased collagen deposition. For these reasons, the skin thickens and it is impossible to pinch it in a normal skin fold. In SSc patients, skin biopsies reveal increased thickness of the dermis and increased amount of collagen deposition, and the disease usually begins with finger involvement in a centripetal pattern. Skin thickening is universal in SSc and generally required for a definitive diagnosis with some exceptions [116,117,118,119]. There are many candidate biomarkers for skin disease in SSc. In skin biopsies, the expression levels of TGF-β1, TGF-βR1 and TGF-βR2 are higher in SSc patients than in healthy subjects [134,135,136,137,138,139,140]. Cutaneous gene expression of macrophage-associated biomarkers (CD14, IL-13RA1) and TGF-β-associated biomarkers (OSMR SERPINE1, CTGF) is associated with cutaneous disease progression in SSc [134,135,136,137,138,139,140]. Marginal zone protein B1 (MZB1) appears to be a good biomarker for cutaneous fibrosis [116,117,118,119]. Circulating levels of periostin are elevated in SSc with extensive cutaneous fibrosis [116,117,118,119]. Sirtuins are NAD-dependent protein deacetylases that regulate angiogenesis; SIRT1 and SIRT3 correlate with the degree of cutaneous fibrosis of the SSc [116,117,118,119]. Adiponectin is reduced in skin affected by SSc [134,135,136,137,138,139,140]. The fibrillar collagen molecule COL4A1, the matricellular protein COMP, the gene encoding spondin-SPON1, and TNC, another ECM protein, have recently been upregulated in the skin of SSc individuals, and all these molecules completely distinguish the normal-skinned SSc [116,117,118,119]. Other investigators found that the upregulated genes IL-13RA1, OSMR and SERPINE 1 were the most predictive of progressive skin disease [134,135,136,137,138,139,140].

Skin thickness in SSc often spontaneously improves over time, confounding many interventional studies, as recently reported with belimumab and nintedanib [184,185,186,187,188,189,190,191,192]. However, mycophenolate, cyclophosphamide, and methotrexate have been shown to improve the mRss and reduce skin thickness in SSc [184,185,186,187,188,189,190,191,192]. Methotrexate has always been problematic in SSc as it can occasionally cause lung inflammation that can be confused with SSc-ILD [184,185,186,187,188,189,190,191,192]. Recently, tofacitinib, although not approved for SSc, has been shown to be more effective than methotrexate in reducing the mRss, ultrasound skin thickness, and musculoskeletal symptoms, and in reducing the biomarker genes regulated by the interferon in SSc [184,185,186,187,188,189,190,191,192]. Ziritaxestat is a selective inhibitor of small autotaxin and reduced mRss in SSc, and thus is a promising new agent currently in clinical trials [184,185,186,187,188,189,190,191,192]. Recently, Fukasawa and colleagues realized the first single-center trial that assessed the pharmacokinetics, safety and efficacy of brodalumab, in Japanese patients with SSc. Brodalumab is a fully human anti-IL-17 receptor A monoclonal antibody. Interestingly, all study patients presented a significant decrease in mRSS, with a reduction in dermal thickness. The authors speculated that, as IL-17 promotes fibroblast proliferation and collagen production, brodalumab might decrease dermal thickness and mRSS by directly inhibiting IL-17 action on fibroblasts [185]. In conclusions, several studies demonstrated that SSc is a Th17-dominant disease in Treg/ Th17 balance and the inhibition of the indirect effects of IL-17 on T and B cell subsets correlated by brodalumab may ameliorate fibrotic skin lesions [184,185,186,187,188,189,190,191,193,194].

## 7. Biomarkers in the Gastrointestinal Systemic Sclerosis Impairment

Gastrointestinal (GI) involvement can be found in approximately 80% of SSc patients (in both the lcSS subgroup and the dcSSc subgroup) and represents a severe manifestation of the disease. All tracts of the gastrointestinal system can be affected, from microstomia to gastroesophageal reflux and gastrointestinal dysmotility to intestinal pseudo-obstruction and fecal incontinence [43,195,196,197,198]. Gastrointestinal involvement may be present from the early stage of the disease and may be asymptomatic making its diagnosis difficult [43,195,196,197,198]. Gastrointestinal system involvement in SSc is profound and includes intestinal and esophageal dysmotility and fibrosis, intestinal ischemia, primary sclerosing cholangitis, primary biliary cirrhosis, bacterial overgrowth, increase in intestinal malignancies, and intestinal inflammation, among other complications [43,195,196,197,198]. Approximately 50% of SSc patients complain of symptoms due to gastric involvement with early satiety, postprandial fullness, bloating, nausea, and epigastric pain. Another complication is represented by the so-called “watermelon stomach” due to microvascular ectasias in the stomach which lead to microhemorrhages and chronic anemia. Patients with SSc may also present with intestinal malabsorption due to mucosal surface reduction and bacterial overgrowth, and small bowel involvement may also manifest as intestinal pseudo-obstruction [43,195,196,197,198]. Furthermore, fecal incontinence is frequent among SSc patients. Treatment of gastrointestinal complications typically focuses on individual problems of gastroesophageal reflux (proton pump inhibitors, H2-blockers, sucralfate), stenosis (dilatation), dysmotility, and bacterial overgrowth (erythromycin, azithromycin, metoclopramide, domperidone, cisapride) [43,195,196,197,198]. In the line of biomarkers, there are elevated fecal levels of the inflammatory biomarker calprotectin in SSc, suggesting that fecal calprotectin could be an effective biomarker for intestinal disease [43,195,196,197,198].

## 8. Biomarkers in Systemic Sclerosis Renal Disease

Scleroderma renal crisis (CRS) is the most frequent renal complication in SSc representing a medical emergency [19,141,142,143,144,145,146,147,148,149,150]. The use of ACE inhibitors has reduced the occurrence of CRS. CRS is characterized by malignant hypertension, microangiopathic haemolysis, microthrombosis, thrombocytopenia, vasospasm, and progressive renal failure which can be caused by a variety of causes, such as various drugs (e.g., corticosteroids, cyclosporine, and tacrolimus) [19,141,142,143,144,145,146,147,148,149,150]. Pathologically, CRS is characterized by rather bland or subtle findings but may show the typical “onion bulb” findings, hyperplasia of the juxtaglomerular apparatus, membranous proliferation, renovascular endothelial damage, intimal proliferation, thrombotic angiopathy, microthrombi of fibrin, hemolysis, vasospasm, vascular occlusion, ischemia, necrosis, vascular remodeling and possibly fibrosis associated with hyperreninemia and accelerated hypertension [19,141,142,143,144,145,146,147,148,149,150]. Anti-fibrillarin antibodies, anti-RNA polymerase III antibodies, and speckled pattern ANA have been closely associated with the development of SRD; however, anti-topoisomerase antibodies have also been associated with a high incidence of CRS in some populations [19,141,142,143,144,145,146,147,148,149,150]. Antiphospholipid antibodies, especially IgG antiphospholipid antibodies, represent a significant risk factor for renal disease in SSc compared with antibody-negative patients [19,141,142,143,144,145,146,147,148,149,150]. Autoantibodies against methionine sulfoxide reductase A, an enzyme important in antioxidant pathways, have been associated with the development of renal and cardiac disease in SSc [19,141,142,143,144,145,146,147,148,149,150].

Biomarkers of CRS include hypertension, elevated uric acid, decreased renal function, thrombocytopenia, hemolytic anemia, and elevated levels of serum soluble CD147 and CD163, renin, mannose-binding lectin, endothelin-1, soluble vascular adhesion molecules, E- selectin, lipocalin-2, angiogenin, apelin, chemerin, complement components and NT-proBNP levels [116,117,118,119,199]. High amounts of serum uric acid, a purine metabolite, may be associated with inflammation, endothelial dysfunction, and renal dysfunction [19,141,142,143,144,145,146,147,148,149,150]. Giant et al. demonstrated in SSc that uric acid is significantly associated with serum creatinine, renal artery resistivity, and decreased glomeruli filtration rate in SSc patients [141,142]. Elevated serum soluble CD147 (sCD147), an inhibitor of extracellular matrix metalloproteinase, and CD163 (sCD163), a cysteine-rich scavenger receptor, have been demonstrated in patients with CRS [19,141,142,143,144,145,146,147,148,149,150]. Similarly, increased endothelin-1 levels and endothelin receptor transport have been associated with CSR [19,141,142,143,144,145,146,147,148,149,150]. Furthermore, soluble vascular adhesion molecules (VCAM-1) and soluble E-selectin have been associated with SRC [19,141,142,143,144,145,146,147,148,149,150]. NT-proBNP is a useful biomarker for CRS and predicts dialysis needs and renal outcomes [116,117,118,119]. CXCL10 is an IFN-inducible chemokine and a potent chemoattractant for Th1 cells and is found to be elevated in CRS patients [19,141,142,143,144,145,146,147,148,149,150]. IL-17B is specifically increased in SSc with renal abnormalities compared to those without [116,117,118,119]. Treatment of CRS focuses on early diagnosis of CRS, prompt use of angiotensin-converting enzyme inhibitors, dialysis, and other supportive measures in anticipation of recovery of renal function [19,141,142,143,144,145,146,147,148,149,150].

## 9. Conclusions

The field of biomarkers in SSc continues to expand in scope and complexity. The sheer number of molecules, pathways, and receptors involved in the pathology of SSc reflect the many complexities of the disease. Several biomarkers are intimately involved in both the pathogenesis and characteristics of the disorder. A biomarker, as broadly defined by the National Institutes of Health Working Group on Biomarkers, is a measure that can be applied for purposes as varied as disease diagnosis, staging, prognostication, measuring or predicting treatment response, and even defining surrogate outcomes [199]. Biomarkers are likely to be of increasing importance for early diagnosis, assessment of disease course and activity monitoring, as well as therapeutic responses, in SSc patients. Furthermore, the introduction of genomics, proteomics, and metabolomics is deepening our understand-ing of the pathophysiology and architecture of SSc. With these developments, a breadth of candidate biomarkers are being studied but the challenge lies in finding readily measurable biomarker which offer specific diagnostic and prognostic value above that of the actually imaging and functional techniques. The numerous environmental triggers and epigenetic mechanisms involved in the pathogenesis of SSc make finding a single biomarker which can accurately represent SSc a further challenge. Biomarkers in scleroderma are being developed to inform overall prognosis, predict treatment response, and quantify outcomes which had been previously defined only clinically. Individual biomarkers such as CXCL4, adiponectin, and CCL18 are demonstrating prognostic utility for fibrotic and vascular complications to an extent they may be clinically useful. Furthermore, N-terminal-pro-BNP and BNP have a well-described association with PAH and systolic pulmonary arterial pressure in SSc, thought to reflect the increasing stress on the right ventricle with increasing pulmonary pressures, and subsequent studies have demonstrated a prospective utility of pro-BNP levels for later development of PAH. Overexpression of type I IFN, TGF-β, PPAR-γ, PI3K-Akt, as well as serum levels of adiponectin, MMP-9, MMP-12, LOX, ADAM12, THBS1, COMP could be used as potential biomarkers of SSc-related skin fibrosis [40,41,42,43,44,45,46,47,76,77,78,79,80,81,82,83,84,85,86,87]. Moreover, recent studies stressed importance of genetic pathway dependent on TGF-β and serum levels of IL-6, KL-6, SP-D, and CCL18 as prominent biomarkers for assessing the severity of fibrosis in SSc-related ILD. In the line of biomarkers, there are elevated fecal levels of the inflammatory biomarker calprotectin in SSc, suggesting that fecal calprotectin could be an effective biomarker for intestinal disease. The most important biomarkers of scleroderma renal crisis include increased levels of serum soluble CD147 and CD163, renin, mannose-binding lectin, endothelin-1, soluble vascular adhesion molecules, E- selectin, lipocalin-2, angiogenin, apelin, chemerin, complement components and NT-proBNP levels. However, with the exception of autoantibodies, today there are no routinely measured biomarkers in SSc and reliable validation of the many potential biomarkers is lacking [40,41,42,43,44,45,46,47,76,77,78,79,80,81,82,83,84,85,86,87,102,103,104,105,106,107,108,109,110]. Several studies shown that specific autoantibodies are important serologic markers for determination subclasses and clinical features of SSc. ANA might not only represent biomarkers of disease but also play a pathogenic role through immune-mediated mechanisms and molecular mimicry. Moreover, the presence of ANA, NVC abnormalities and recurrent Raynaud’s phenomenon are predictive factors for progression to definitive SSc. In particular, anti-topoisomerase I antibodies are predictive, in the first 3 years of disease of the development of diffused skin involvement and digital ulcers (DU), as well as severe interstitial lung disease (ILD) [65,66,67,68,69,70,71,72,73,74,76,77,78,79,80,81,82,83,84,85,86,87,119,120,121,122,123,124,125,126,127]. The anti-centromere autoantibodies (ACA) are associated with PAH and anti-topoisomerase I autoantibodies with ILD. Finally, anti-RNA polymerase III autoantibodies may be biomarkers for rapid progression of skin thickening, gastric antral vascular ectasia, SSc-associated tumors and scleroderma renal crisis. In summary, identifying biomarkers, which can offer diagnostic and prognostic certainty, may help SSc patients to receive preventative treatment as part of a personalised medicine approach. Finally, large randomised controlled trials, which have facilitated new licensed treatments in SSc, have also offered valuable insight into the response of candidate biomarkers but further large scale studies focussing on biomarkers are needed to validate these in order to incorporate them into routine disease stratification.

## Figures and Tables

**Table 1:** Correlation between autoantibodies, clinical manifestations and skin subset.

Autoantibodies	Frequency %	Subset	Clinical Associations
Anti-Topo I	15–25%	dcSSc	Cardiac, skin and lung involvement
ACA	10–20%	lcSSc	CREST, DU, PAH
Anti-RNA Pol III	10–25%	dcSSc	SRC, tendon friction rubs, cardiac involvement
Anti-U1 RNP	5–30%	lcSSc	MCTD
Anti-PMScl	3–8%	Myositis SSc	Inflammatory muscle involvement
Anti-To/To	2–5%	lcSSc	PAH

Legend. Autoantibodies in SSc and their correlations with skin subset and clinical manifestations; CREST: Calcinosis, Raynaud’s phenomenon, Esophageal dysmotility, Sclerodactyly, and Teleangiectasia; DU: digital ulcers; PAH: pulmonary hypertension¸ SRC: scleroderma renal crisis; MCTD: mixed connective tissue disease.

**Table 2:** Biomarkers in systemic sclerosis.

Biomarker	Clinical Association
IL-6↑ [6,81,82,83,84,85,86,87,88,89,95,96,97,98,101,108]	mRSS, early progressive skin sclerosis, poor prognosis, DLco decline in SSc-ILD
CCL2↑ [30,31,32,33,34,35,36,37,38,39,40,41,42,59,60,61,62,63,64,65,66,67,81,82,83,84,85,86,87]	ILD (lung dysfunction, CT scores), mRSS
CTGF↑ [81,82,83,84,85,86,87]	mRSS, ILD
CXCL4↑ [81,82,83,84,85,86,87,116,117,118,119]	mRSS, lung fibrosis, PAH, disease progression
CX3CL1↑ [68,81,82,83,84,85,86,87]	dcSSc, ILD, digital ulcer
ICAM-1↑ [84,85,86,87,88,89,90]	Rapidly progressive disease, digital ulcers, dcSSc, ILD, joint involvement, renal crisis, predictive of respiratory dysfunction
Von Willebrand factor↑ [118,119,120,121,122,123,124,125,126,127,128,129,130,131,132,133]	Raynaud’s phenomenon, disease severity, ILD, predictive of PAH
SP-D↑ [81,82,83,84,85,86,87,88,89,90]	Severity of ILD, maximum fibrosis scores on HRCT
CCL18↑ [81,82,83,84,85,86,87]	Activity and severity of ILD, predictive worsening of ILD and mortality
MMP-7↑ [2,29,81,82,83,84,85,86,87]	ILD, disease severity
MMP-12↑ [2,29,81,82,83,84,85,86,87,116,117,118,119]	Skin sclerosis, dcSSc, ILD, nailfold bleeding, lower FVC
CRP↑ [2,10,41,42,43,44,45,46,47,48,49,50,114,115,116,117,118,119,120]	Skin sclerosis, PAH, renal dysfunction, risk of progressive early ILD, worse pulmonary function
TGF-β↑ [81,82,83,84,85,86,87,116,117,118,119,134,135,136,137,138,139,140]	Digital ulcers, dcSSc
TGF-β↓ [81,82,83,84,85,86,87,116,117,118,119,134,135,136,137,138,139,140]	dcSSc, mRSS (in dcSSc)
VEGF↑ [6,88,89,95,96,97,98,100,101,102,103,104,105,106,107,108,116,117,118,119]	Systemic organ involvement, PAH, shorter disease duration, skin sclerosis, reduced capillary density of nailfold
VEGF↓ [6,88,89,95,96,97,98,100,101,102,103,104,105,106,107,108,116,117,118,119]	Digital ulcers
CXCL8↑ [81,82,83,84,85,86,87]	Predictive of physical dysfunction
CXCL10↑ [19,81,82,83,84,85,86,87,141,142,143,144,145,146,147,148,149,150]	Preclinical/early SSc
VCAM-1↑ [19,141,142,143,144,145,146,147,148,149,150]	Systemic organ involvement, renal crisis, disease activity
E-selectin↑ [19,116,117,118,119,141,142,143,144,145,146,147,148,149,150]	Systemic organ involvement, renal crisis, disease activity
P-selectin↑ [19,116,117,118,119,141,142,143,144,145,146,147,148,149,150]	Disease activity, predictive of physical disability
Endostatin↑ [38,62,63,64,65,66,67,116,117,118,119]	PAH
BNP/NT pro-BNP↑ [116,117,118,119,120,121,122,123,124,125,126,151]	Severity, stability, and prognosis of PAH
Endothelin-1↑ [16,17,18,19,20,38,39,40,41,42,43,44,45,116,117,118,119,120,121,122,123,124,125,126,127,128,129,130,131,132,133,141,142,143,144,145,146,147,148,149,150,151,152,153]	PAH, systemic organ involvement, microangiopathy defined by capillaroscopy
Type I collagen (C-terminal telopeptide)↑ [79,80,81,82,83,84,85,86,87,88,89,90,91,92,93,94,116,117,118,119]	Skin fibrosis, mRSS, pulmonary dysfunction, CRP
Type III collagen (N-terminal peptide)↑ [79,80,81,82,83,84,85,86,87,88,89,90,91,92,93,94,116,117,118,119]	Disease activity, mRSS, HRCT score, prognosis
MMP-9↑ [2,29,116,117,118,119]	mRSS, dcSSc
MMP-12↑ [2,29,81,82,83,84,85,86,87,116,117,118,119]	Skin sclerosis, dcSSc, ILD, nailfold bleeding, lower FVC

Legend. ↑, upregulated; ↓, downregulated; IL-6, interleukin 6; CTGF, connective tissue growth factor; ICAM-1, intercellular adhesion molecule 1; SP-D, surfactant protein-D; MMP, matrix metalloproteinases; TGF-β, transforming growth factor; VEGF, vascular endothelial growth factor; NT-proBNP, N-terminal-pro hormone BNP; ILD, interstitial lung disease; DLco, diffusing capacity of carbon monoxide; CT, computed tomography; PAH, pulmonary arterial hypertension; HRCT, high resolution CT; mRSS, modified Rodnan total skin thickness score; dcSSc, diffuse cutaneous systemic sclerosis; lcSSc, limited cutaneous systemic sclerosis.

## Data Availability

All the data are available upon reasonable request to the corresponding author.
